# Clinical Impact of Linezolid Therapeutic Drug Monitoring on the Tolerability of Prolonged Courses in the Outpatient Setting

**DOI:** 10.1093/ofid/ofag318

**Published:** 2026-05-21

**Authors:** Jeffrey M Harrington, Jerod L Nagel, Samantha Sallerson, Xuping Yan, Samuel L Aitken, Vincent D Marshall, Beth Lawless, Manjunath P Pai, Jason M Pogue, Gregory Eschenauer

**Affiliations:** Department of Pharmacy, Michigan Medicine, Ann Arbor, Michigan, USA; Department of Clinical Pharmacy, University of Michigan College of Pharmacy, Ann Arbor, Michigan, USA; Department of Pharmacy, Michigan Medicine, Ann Arbor, Michigan, USA; Department of Pharmacy, Michigan Medicine, Ann Arbor, Michigan, USA; Department of Pharmacy, Michigan Medicine, Ann Arbor, Michigan, USA; Department of Pharmacy, Michigan Medicine, Ann Arbor, Michigan, USA; Department of Clinical Pharmacy, University of Michigan College of Pharmacy, Ann Arbor, Michigan, USA; Division of Infectious Diseases, University of Michigan Medical School, Ann Arbor, Michigan, USA; Department of Pharmacy, Michigan Medicine, Ann Arbor, Michigan, USA; Department of Clinical Pharmacy, University of Michigan College of Pharmacy, Ann Arbor, Michigan, USA; Department of Pathology, Michigan Medicine, Ann Arbor, Michigan, USA; Department of Pharmacy, Michigan Medicine, Ann Arbor, Michigan, USA; Department of Clinical Pharmacy, University of Michigan College of Pharmacy, Ann Arbor, Michigan, USA; Department of Pharmacy, Michigan Medicine, Ann Arbor, Michigan, USA; Department of Clinical Pharmacy, University of Michigan College of Pharmacy, Ann Arbor, Michigan, USA; Department of Pharmacy, Michigan Medicine, Ann Arbor, Michigan, USA; Department of Clinical Pharmacy, University of Michigan College of Pharmacy, Ann Arbor, Michigan, USA

**Keywords:** complex infections, linezolid, outpatient, therapeutic drug monitoring, thrombocytopenia

## Abstract

**Background:**

Linezolid is a gram-positive oral antibiotic whose duration-dependent risk of thrombocytopenia has historically constrained durations of therapy to <3 weeks. This study aimed to evaluate whether therapeutic drug monitoring (TDM)–based management of linezolid can support prolonging the course of therapy for complex infections.

**Methods:**

This single-center, retrospective cohort study included patients ≥18 years old discharged on linezolid for a planned duration of ≥3 weeks between January 1, 2023, and April 1, 2025. The primary outcome was early discontinuation of linezolid due to thrombocytopenia, compared between patients managed with TDM and matched controls without TDM, using 1:1 matching based on planned treatment duration.

**Results:**

One hundred sixty-two patients were included, with 81 in each study arm. The mean duration of linezolid (SD) was 32.1 (12.4) days, and 100 (62%) patients were treated for bone and joint infections. Patients receiving TDM were significantly more likely to be age ≥65 years, have recent surgery, receive Complex Outpatient Antimicrobial Therapy (COpAT) monitoring, have longer inpatient courses, and have additional laboratory monitoring performed. The primary outcome of early discontinuation from thrombocytopenia occurred in 11 (14%) patients vs 25 (31%) patients in the TDM and control arms, respectively (adjusted odds ratio, 0.07; 95% CI, 0.02–0.26). Thrombocytopenia remained the most common toxicity in both cohorts.

**Conclusions:**

Linezolid TDM was associated with markedly lower rates of early discontinuation from thrombocytopenia, underscoring the potential value of early TDM and selective dose adjustment to enhance tolerability during prolonged treatment courses.

## Teaser

In patients with planned linezolid courses > 3 weeks, TDM reduced early discontinuation due to thrombocytopenia. Thrombocytopenia remained the predominant toxicity. Early TDM may improve tolerability and facilitate prolonged therapy for complex infections.

Linezolid is an oxazolidinone antibiotic with excellent in vitro activity against gram-positive organisms including methicillin-resistant *Staphylococcus aureus* and vancomycin-resistance *Enterococcus faecium* [1, 2]. The pharmacokinetic parameters of linezolid are also favorable, with nearly 100% bioavailability and tissue distribution to sites where it is traditionally difficult to achieve high drug exposures, such as the lungs, bones, and cerebrospinal fluid [3–5]. These characteristics make linezolid an attractive oral option for outpatient management of complex infections by circumventing the need for central line access and daily infusions and thus potentially improving patient satisfaction, avoiding the risk of central line–associated complications, and decreasing health care costs.

Unfortunately, the role of linezolid in this setting has been limited due to toxicities associated with prolonged durations of therapy. In particular, significant thrombocytopenia can occur with durations longer than 10–14 days [6]. It has been proposed that this is caused by mitochondrial toxicity, as bacterial and mitochondrial ribosomes possess structural similarities and common binding sites, resulting in inhibition of mitochondrial protein synthesis and impairment of megakaryocyte differentiation [7, 8]. However, the observed duration-dependent effect of linezolid on thrombocytopenia may at least be partially explained by elevated drug exposures. Multiple studies have observed a correlation between elevated trough (C_min_) concentrations and thrombocytopenia [9–12]. Taken together, these data are supportive of an exposure threshold above 7–10 mcg/mL being associated with increased risk of thrombocytopenia [13], though variable definitions of thrombocytopenia and lack of representation of patients from the United States limit interpretability.

Given that supratherapeutic linezolid exposures are predictive of thrombocytopenia and significant interpatient pharmacokinetic variability exists, therapeutic drug monitoring (TDM) may offer an effective strategy for dose optimization to improve long-term tolerability. As thrombocytopenia often manifests after several weeks on treatment, early TDM-guided dose adjustments may reduce the likelihood of developing this toxicity [14]. While there are several studies identifying an association between high linezolid C_min_ and thrombocytopenia, there is limited evidence exploring whether TDM-guided dosing improves tolerability and facilitates completion of prolonged treatment courses. Thus, additional evidence to elucidate the clinical benefit of performing TDM in patient populations at risk for toxicities is necessary.

Accordingly, this study was designed to test the hypothesis that application of linezolid TDM in patients with a planned duration of ≥3 weeks reduces the incidence of early drug discontinuation due to thrombocytopenia when compared with a similar group of control patients who did not receive any TDM.

## METHODS

### Study Design and Setting

This matched retrospective cohort analysis was conducted at Michigan Medicine. In October 2023, Michigan Medicine implemented send-out testing of total linezolid plasma concentrations, allowing for viewable results within 48–72 hours. Along with this update, the antimicrobial stewardship program published linezolid TDM guidelines (https://antimicrobialstewardship.med.umich.edu/guidelines/adult/Linezolid-TDM), describing risk factors for toxicity and thus when to consider TDM, goal C_min_ range (2–8 mcg/mL), timing of collection, and appropriate dose adjustments. Importantly, TDM was recommended for all anticipated durations of therapy >2 weeks. TDM was performed at the discretion of an Infectious Diseases pharmacist or physician, and all concentrations were interpreted by an Infectious Diseases pharmacist in both the inpatient and outpatient settings.

### Patient Consent

This study was approved by the Institutional Review Board at the University of Michigan (HUM00269874). Due to the retrospective nature of the study, written informed consent was not required.

### Patient Population

Linezolid courses initiated between January 1, 2023, and April 1, 2025, were screened for eligibility. Eligible patients were aged ≥18 years with a planned linezolid course of ≥3 weeks and receipt of a minimum of 3 days of linezolid. Linezolid therapy must have either started inpatient and continued at hospital discharge or been newly prescribed at the time of hospital discharge. Reasons for exclusion were lack of platelet monitoring performed after discharge, first TDM within 7 days before planned stop date, receipt of myelosuppressive chemotherapy during treatment, any mycobacterial indication for linezolid (as the empirical use of 600-mg once-daily dosing is often utilized), or discontinuation of linezolid before completion of the planned course for a reason definitively unrelated to therapy. If a patient met criteria for multiple linezolid courses, only the first eligible course in the study window was included in the analysis.

### Procedures and Outcomes

Potential TDM cases were identified from a log of all patients with ≥1 sample submitted for linezolid plasma concentration measurement. Controls were identified by screening all patients with both an inpatient linezolid order and a discharge linezolid prescription during the study window, excluding anyone with TDM performed. Eligibility screening continued in chronological order until statistical power was achieved (discussed below) or all identified patients were screened, whichever came first. Eligible patients who received TDM were matched (1:1) with control patients who did not receive TDM by initial planned duration of therapy (21–27 days, 28–41 days, or ≥42 days) to form the matched population. Patients who were unable to be matched by initial planned duration were excluded from the analysis. A subsequent exploratory analysis was performed including patients who met per-protocol (PP) criteria for TDM compared with controls in the matched population. PP TDM was defined as a steady-state linezolid C_min_ obtained within 10 days after treatment initiation, with a total daily dose reduction also occurring within 10 days of treatment initiation for patients with C_min_ ≥8.0 mcg/mL. The PP TDM arm was compared against the original matched control arm without rematching, as both arms remained balanced with respect to planned duration. A linezolid concentration was considered at steady-state C_min_ if the patient received ≥4 doses on a stable dose and frequency and the sample was collected after two-thirds of the current dose interval. Concentrations obtained outpatient were presumed to be steady-state C_min_ unless explicitly documented otherwise. Data on patient demographics, clinical characteristics (including surgical procedures, complex outpatient antimicrobial therapy program [COpAT] enrollment, infection type, baseline and weekly serum creatinine and platelets), hospital discharge date, linezolid start/stop dates, dosing and TDM, adverse events, and clinical outcomes were collected from medical records and managed using REDCap electronic data capture tools hosted at Michigan Medicine [15].

The primary outcome was early discontinuation of linezolid due to thrombocytopenia in the matched population. To meet the primary outcome, thrombocytopenia must have been explicitly noted as a reason for discontinuation in the medical chart, and the primary outcome could still be met if other toxicities were also reported. Early discontinuation was defined as stopping linezolid therapy before the stop date set by Infectious Diseases in their final patient plan. Secondary outcomes included early discontinuation of linezolid due to thrombocytopenia in the PP population, early discontinuation of linezolid for any reason, and development of clinically significant thrombocytopenia, each assessed in both the matched and PP populations. Three definitions of thrombocytopenia were evaluated: 25%, 50%, and 25% with a nadir <150 K/mcL, where percentages refer to the reduction from baseline platelet count at linezolid initiation.

### Statistical Analysis

A sample size of 194 patients was calculated to have an 80% power to detect a 20% difference in the primary outcome between the TDM and control arms, assuming a 60% rate of early discontinuation in the control arm and a fixed α value of .05. The anticipated overall effect size was based on internal data from 18 patients enrolled in the Michigan Medicine COpAT program, which identified 40% and 60% early discontinuation rates in TDM and non-TDM patients, respectively. Between-group differences in baseline characteristics and unadjusted outcomes were compared using chi-square or Fisher exact tests for dichotomous variables, while 2-sided Student *t* and Mann-Whitney *U* tests were used for parametric and non–parametrically distributed continuous data, respectively. Backwards stepwise logistic regression analysis for primary and secondary outcomes was performed, adjusting for clinically relevant variables associated with either the treatment group or study outcome. Results of the logistic regression are described as adjusted odds ratios (ORs) with a 95% confidence interval. All statistical tests were performed in SPSS (version 30).

## RESULTS

### Patient Population

**Figure 1.**
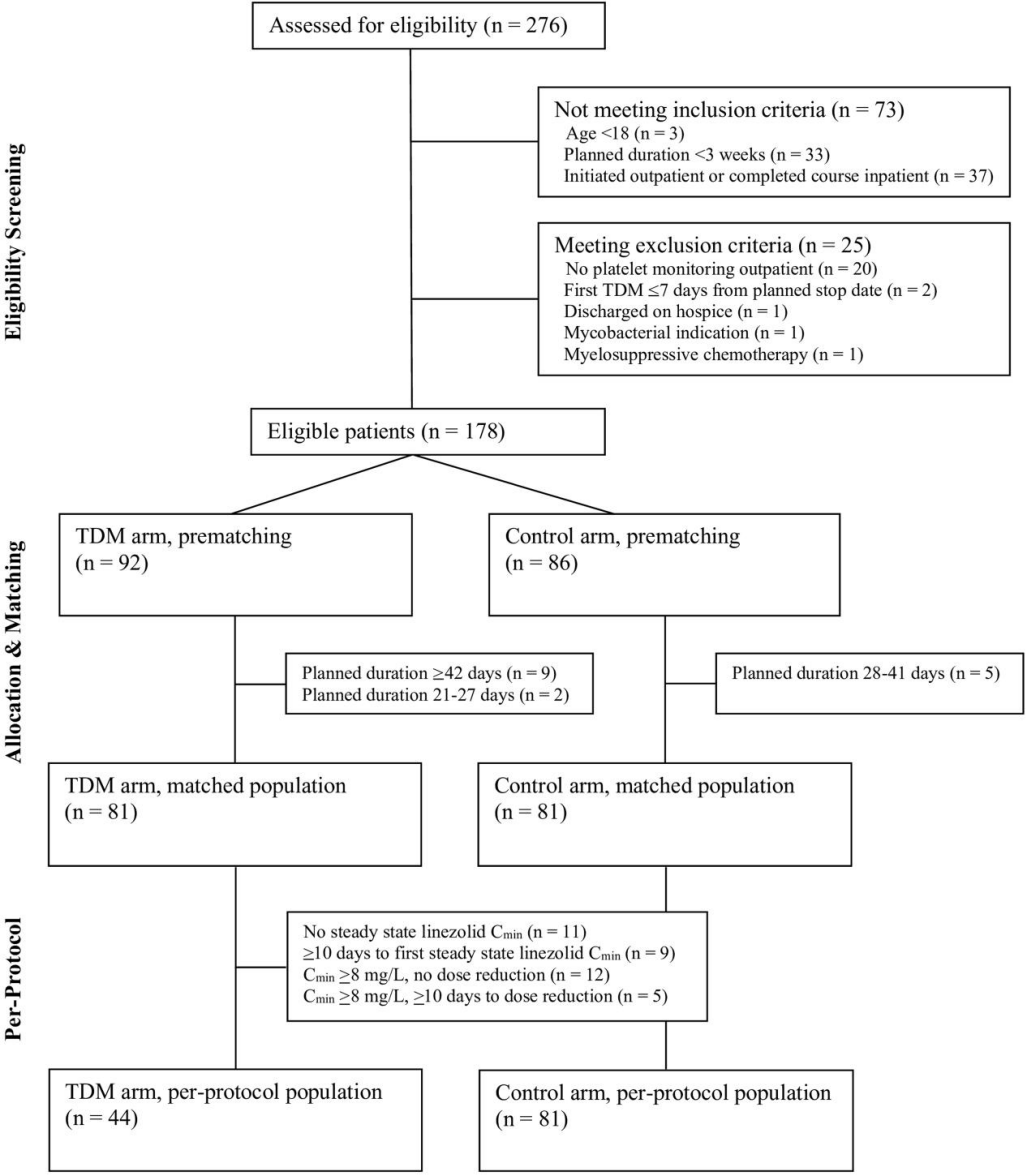
CONSORT diagram. Abbreviation: TDM, therapeutic drug monitoring.

A total of 276 patients prescribed linezolid were screened, of whom 178 were considered eligible. After matching by planned duration, 162 patients (81 in each arm) remained for analysis in the matched population. A detailed record of the study flow, including reasons for exclusion, is presented in Figure 1.

Patient characteristics are summarized in Table 1. The mean age (SD) was 59 (15) years, 57% of patients were male, and the mean body mass index (SD) was 29.1 (7.6) kg/m^2^. The mean baseline platelet count (SD) was 309 (130) K/mcL, and the median (interquartile range [IQR]) creatinine clearance (CrCl) was 87 (60–122) mL/min. The most common indications for linezolid were bone and joint (62%) and skin and soft tissue (18%) infections. The mean total duration of linezolid (SD) was 32.1 (12.4) days, with most courses occurring primarily outpatient. Patients receiving TDM were significantly more likely to be ≥65 years old, have a surgical procedure performed during treatment, be enrolled in COpAT monitoring, have a longer inpatient duration of treatment, and have more outpatient platelet monitoring performed (Table 1).

**Table 1.** Demographics and Baseline Characteristics of Patients Receiving Linezolid in the Matched Population

Characteristic	Total (n = 162)	TDM (n = 81)	Control (n = 81)	*P* Value
Age, y	59 (±15)	61 (±16)	57 (±14)	.15
≥60 y	89 (55)	50 (62)	39 (48)	.08
≥65 y	69 (43)	42 (52)	27 (33)	**.02**
Male	92 (57)	46 (57)	46 (57)	1.00
BMI, kg/m^2^	29.1 (±7.6)	28.6 (±7.4)	29.6 (±7.8)	.43
Serum creatinine, mg/dL	0.83 (0.66–1.18)	0.86 (0.67–1.26)	0.82 (0.66–1.10)	.40
CrCl, mL/min	87 (60–122)	81 (59–104)	91 (62–126)	.19
<60 mL/min	39 (24)	21 (26)	18 (22)	.58
<30 mL/min	6 (4)	4 (5)	2 (2)	.41
Platelet count, K/mcL	309 (±130)	297 (±132)	321 (±128)	.23
Surgical procedure	31 (19)	23 (28)	8 (10)	**.003**
COpAT monitoring	31 (19)	25 (31)	6 (7)	**<.001**
Receipt of other antibiotics	91 (56)	49 (60)	42 (52)	.27
Infection type^a^				
Bone and joint	100 (62)	47 (58)	53 (65)	.33
Skin and soft tissue	29 (18)	14 (17)	15 (19)	.84
Intra-abdominal	15 (9)	7 (9)	8 (10)	1.00
Endovascular	14 (9)	10 (12)	4 (5)	.16
Bloodstream	12 (7)	6 (7)	6 (7)	1.00
Respiratory	4 (3)	3 (4)	1 (1)	.62
Central nervous system	3 (2)	1 (1)	2 (2)	1.00
Other	11 (7)	4 (5)	7 (9)	.35
Initial planned duration of linezolid				
21–27 d	32 (21)	17 (21)	17 (21)	–
28–41 d	104 (64)	52 (64)	52 (64)	–
≥42 d	24 (15)	12 (15)	12 (15)	–
Linezolid duration inpatient, d	4.9 (±5.8)	6.5 (±7.0)	3.4 (±3.7)	**<.001**
Linezolid duration outpatient, d	28.1 (±12.4)	28.1 (±13.9)	28.2 (±10.8)	.96
Total linezolid duration, d	32.1 (±12.4)	33.7 (±13.6)	30.7 (±10.9)	.12
Weekly outpatient CBCs	75 (46)	42 (52)	33 (41)	.16
Total outpatient CBCs drawn	2.8 (±1.7)	3.1 (±1.9)	2.6 (±1.5)	**.05**

Continuous variables are reported as mean (±SD) if parametric and median (IQR) if nonparametric; frequencies are reported as No. (%). Bolded *P*-values indicate a statistically significant result. Demographics and laboratory parameters are reported at the time of linezolid initiation.

Abbreviations: BMI, body mass index; CBC, complete blood count; COpAT, complex outpatient antimicrobial therapy; CrCl, creatinine clearance; TDM, therapeutic drug monitoring.

^a^Multiple infection types may copresent, and they are reported individually (eg, osteomyelitis with overlying cellulitis is both bone and joint and skin and soft tissue).

### Linezolid Dosing and Therapeutic Drug Monitoring

The median initial dose prescribed (IQR) was 1200 (1200–1200) mg/d; 3 patients in the matched population were initiated on linezolid at 600 mg orally (PO) once daily. A total of 29 (36%) patients received a dose reduction in the TDM arm compared with 1 (1%) patient in the control arm (*P* < .001). With the exception of 1 patient on 300 mg PO twice daily, all reduced-dosing regimens were prescribed as 600 mg PO once daily. No patients required a dose increase.

Of the 81 patients in the TDM arm, 70 (86%) had at least 1 linezolid concentration drawn as a steady-state C_min_ during treatment. The median first steady-state C_min_ (IQR) was 9.4 (3.5–13.6) mcg/mL, drawn after a median (IQR) of 3.5 (2–7) days. Remeasurement of a linezolid C_min_ following a dose reduction occurred in 12 (41%) patients. The corresponding pre- and postadjustment steady-state C_min_ for this subgroup (IQR) was 15.7 (11.9–17.9) mcg/mL and 4.2 (2.3–5.3) mcg/mL, respectively.

The first steady-state C_min_ was drawn before day 10 of treatment in 61 (75%) patients. Of these 61 patients, 12 had a steady-state C_min_ ≥8.0 mcg/mL with no dose reduction made, and an additional 5 patients with steady-state C_min_ ≥8.0 mcg/mL had a dose reduction take place on or after day 10. Thus, 44 patients in the TDM arm remained in the PP population. Baseline characteristics of this population and differences between PP and non-PP TDM are described in Supplementary Tables 1 and 2, respectively.

### Outcomes

The primary outcome of early discontinuation due to thrombocytopenia was significantly less likely in the TDM arm compared with the control arm in the matched population, occurring in 11 (14%) and 25 (31%) patients in their respective arms (unadjusted OR, 0.35; 95% CI, 0.16–0.78) (Table 2). Controlling for baseline CrCl, COpAT monitoring, endovascular infection, and total duration of therapy, TDM was independently associated with lower odds of the primary outcome (adjusted OR, 0.07; 95% CI, 0.02–0.26). Time to early discontinuation due to thrombocytopenia was similar between study arms, occurring at a mean (SD) of 20.1 (5.6) days and 23.0 (5.4) days in the TDM and control arms, respectively (*P* = .15). Limiting the assessment to the PP population, the corresponding rates of early discontinuation due to thrombocytopenia were 7 (16%) patients in the TDM arm and 25 (31%) patients in the control arm (unadjusted OR, 0.42; 95% CI, 0.17–1.08). After adjustment for confounders, TDM was associated with lower odds of early discontinuation due to thrombocytopenia in this subgroup (adjusted OR, 0.07; 95% CI, 0.01–0.42).

**Table 2.** Outcomes by Use of Linezolid TDM in the Matched and PP Populations

Outcome	Total	TDM	Control	Unadjusted OR	*P* Value	Adjusted OR	*P* Value
Matched population	n = 162	n = 81	n = 81	…		**…**	**…**
Early discontinuation due to thrombocytopenia^e,a^	36 (22)	11 (14)	25 (31)	0.35 (0.16–0.78)	**.008**	0.07 (0.02–0.26)	**< .001**
Total early discontinuations^b^	57 (35)	25 (31)	32 (40)	0.68 (0.36–1.31)	.25	0.23 (0.08–0.67)	**.007**
25% platelet reduction	111 (69)	53 (65)	58 (72)	0.76 (0.39–1.49)	.40	0.68 (0.31–1.52)	.35
50% platelet reduction	52 (32)	20 (25)	32 (40)	0.50 (0.26–0.98)	**.04**	0.39 (0.17–0.94)	**.04**
25% platelet reduction and <150 K/mcL	54 (33)	24 (30)	30 (37)	0.72 (0.37–1.38)	.32	0.39 (0.16–0.94)	**.04**
PP population	n = 125	n = 44	n = 81	…		…	…
Early discontinuation due to thrombocytopenia^c^	32 (26)	7 (16)	25 (31)	0.42 (0.17–1.08)	.07	0.07 (0.01–0.42)	**.003**
Total early discontinuations^d^	49 (39)	17 (39)	32 (40)	0.96 (0.45–2.05)	.93	0.28 (0.07–1.07)	.06
25% platelet reduction	87 (70)	29 (69)	58 (72)	0.78 (0.35–1.72)	.51	0.60 (0.21–1.70)	.34
50% platelet reduction	46 (37)	14 (32)	32 (40)	0.72 (0.33–1.55)	.40	0.55 (0.20–1.51)	.24
25% platelet reduction and <150 K/mcL	42 (34)	12 (27)	30 (37)	0.64 (0.29–1.42)	.27	0.31 (0.10–0.99)	**.05**

Frequencies are reported as No. (%); ORs are reported with 95% CI. Bolded *P*-values indicate a statistically significant result. Adjusted ORs were calculated by controlling for variables in backwards stepwise conditional logistic regression. Variables in the final model are reported below for key outcomes.

Abbreviations: CBC, complete blood count; COpAT, complex outpatient antimicrobial therapy; CrCl, creatinine clearance; OR, odds ratio; PP, per-protocol; TDM, therapeutic drug monitoring.

^a^Controlled for baseline CrCl, COpAT monitoring, endovascular infection, and total duration of therapy.

^b^Controlled for COpAT monitoring, endovascular infection, surgical procedures, total duration of therapy, and weekly outpatient CBCs.

^c^Controlled for COpAT monitoring, endovascular infection, and total duration of therapy.

^d^Controlled for COpAT monitoring, endovascular infection, surgical procedures, and total duration of therapy.

^e^Nonthrombocytopenia adverse events were coreported in n = 3 and n = 5 patients in the TDM and control arms, respectively.

Development of thrombocytopenia was common in the study, observed in 111 (69%), 52 (32%), and 54 (33%) patients in the overall matched population at thresholds of 25% platelet reduction, 50% platelet reduction, and 25% with platelet nadir <150 K/mcL, respectively (Table 2). Thrombocytopenia was also the most frequent cause of early discontinuation, but other factors accounted for additional discontinuation. Early discontinuation for any reason was observed in 25 (31%) patients in the TDM arm and 32 (40%) patients in the control arm (unadjusted OR, 0.68; 95% CI, 0.36–1.31). TDM was associated with lower odds of this outcome after adjustment (adjusted OR, 0.23; 95% CI, 0.08–0.67). Individual (nonthrombocytopenia) events leading to early discontinuation, as well as a full description of all patient-reported adverse events in the study, can be found in Supplementary Tables 3 and 4 , respectively.

## DISCUSSION

An increasing body of randomized controlled trial evidence evaluating oral therapies for complex orthopedic and endovascular infections has solidified their role as efficacious alternatives to intravenous therapies [16, 17]. While linezolid offers a particularly attractive option in this setting, thrombocytopenia risk has limited its uptake. Performing linezolid TDM has been proposed to improve tolerability, but data to inform TDM implementation and dose adjustments in clinical practice or establishing a direct clinical benefit of such a practice remain scarce. In this single-center, matched, retrospective cohort study, we found that the use of TDM, primarily via assessment of a single linezolid C_min_ per patient, significantly reduced the likelihood of early therapy discontinuation directly attributable to thrombocytopenia compared with patients who did not receive TDM.

Our findings suggest a role for TDM in patients anticipated to receive prolonged courses of linezolid. To our knowledge, only 1 other study has assessed the impact of linezolid TDM on safety outcomes. LIMMIT1 was a multicenter retrospective analysis of 622 patients in Australia that received >5 days of linezolid, of which <25% underwent linezolid TDM [14]. The investigators found that performance of TDM was independently associated with the development of toxicity; however, TDM-guided “appropriate dose adjustment” based on the first concentration obtained was protective against toxicity. Interpreting these contradictory findings is challenging. On one hand, patients who received TDM had lower baseline platelet counts, higher baseline creatinine, and longer durations of therapy compared with those without, which may explain why TDM was associated with increased toxicity. On the other hand, “appropriate dose adjustment” included patients who were therapeutic and did not need dose modification, as well those who had dose increases due to subtherapeutic concentrations. While the number of patients requiring dose increases was not described, 35/70 (50%) of those reported with “appropriate dose adjustments” did not require dose adjustments due to therapeutic concentrations at the time of TDM, and thus this subgroup may have experienced less toxicity due to being largely represented by those with concentrations below toxicity thresholds. Taken together, it is unclear what impact TDM had on avoiding overexposure and rates of toxicity in this study. This is further complicated by the fact that the end point of toxicity was not explicitly restricted to thrombocytopenia, and details about which toxicities were experienced are not available. In contrast, TDM in the present analysis was performed in the context of institutional guidelines recommending proactive TDM, this cohort was evenly distributed between those with and without TDM and matched based on planned duration of therapy, and our primary end point was specific to thrombocytopenia, the toxicity of greatest concern in this population.

Given the observational nature of the study and variable implementation of TDM with respect to timing of concentrations and dose adjustments, an exploratory PP subgroup analysis was planned to evaluate outcomes in the clinical context of what was considered “optimal” TDM. The PP subgroup definition was chosen on the premise that the risk of linezolid-induced thrombocytopenia has been consistently observed to increase significantly after 10–14 days of treatment [18–22]. A maximum of 10 days for linezolid C_min_ assessments and dose adjustments following initiation would thus allow for supratherapeutic exposures to be addressed before the anticipated onset of thrombocytopenia. This analysis is limited due to the smaller sample size of the PP cohort. Reassuringly, however, TDM remained protective against early discontinuation due to thrombocytopenia in the PP subgroup.

The relatively higher frequency of COpAT monitoring among patients who received TDM compared with controls is a key imbalance with implications on our overall findings. Patients enrolled into the COpAT program at hospital discharge receive intensive safety monitoring, coordination of labs and follow-up, and therapy plan assessments and adjustments via weekly follow-up with a dedicated pharmacist. Initiation of the COpAT program at Michigan Medicine also coincided with changes to internal stewardship guidelines encouraging increased use of oral linezolid (as an alternative to intravenous therapies) in complex scenarios, most notably for osteomyelitis and native valve endocarditis. Accordingly, as the distribution of other baseline characteristics (eg, age ≥65, surgical procedures, and endovascular infections) would indicate, patients who may not have previously been deemed candidates for oral therapy were being prescribed linezolid with reliance on TDM, intensive platelet monitoring, and COpAT management to improve clinical success. These consistent differences in baseline characteristics may have biased against the TDM arm with respect to the primary outcome. In addition, COpAT monitoring with consistent, detailed pharmacist–patient interviews creates more opportunities to identify toxicities, which may explain the higher frequency of discontinuation for nonthrombocytopenia adverse events in the TDM arm.

There are important limitations to consider with this work. As this study was retrospective in nature, there was a reliance on the adequacy and clarity of medical record documentation from outpatient visits to assign the primary outcome. However, our primary outcome finding was validated by our use of objective secondary end points. The single-center aspect of the study design also has implications for the external validity of our findings, which could vary if other sites implement TDM with slower result turnaround time or reduced capacity for outpatient follow-up. Additionally, given the relatively limited sample size, we were only capable of matching by planned duration of therapy while maintaining adequate study power. Availability of more eligible patients would enable matching by other potential confounding variables, such as age, infection type, or COpAT monitoring. Finally, directly evaluating clinical success was not a goal of this study. Our study population of relatively small numbers of diverse infection types, collected in a retrospective fashion, is not conducive to such an approach. For similar reasons, we were unable to assess the impact of linezolid TDM on neurotoxicity, and the role of TDM in limiting neurotoxicity remains unknown.

## CONCLUSIONS

In this study of patients discharged on prolonged (≥3-week) courses of linezolid, TDM was associated with markedly lower rates of early discontinuation due to thrombocytopenia. Dose adjustments were frequently performed following TDM, but almost never occurred without TDM. These findings are supportive of early TDM and subsequent dose adjustments to improve the tolerability of linezolid when prolonged courses are anticipated. Larger prospective studies are warranted to further explore the impact of TDM-guided linezolid therapy on clinical outcomes.

## Supplementary Material

ofag318_Supplementary_Data

## Supplementary Data

Supplementary materials are available at *Open Forum Infectious Diseases* online. Consisting of data provided by the authors to benefit the reader, the posted materials are not copyedited and are the sole responsibility of the authors, so questions or comments should be addressed to the corresponding author.
