# Supplementary material for: Clinical Impact of Linezolid Therapeutic Drug Monitoring on the Tolerability of Prolonged Courses in the Outpatient Setting
Source: Open Forum Infect Dis. 2026 May 21;13(6):ofag318. doi: 10.1093/ofid/ofag318 (PMC13243916; doi:10.1093/ofid/ofag318)
Supplement: ofag318_Supplementary_Data [file ofag318_supplementary_data.doc]

**Table S1.** Demographics and baseline characteristics of patients receiving linezolid in the PP population

| **Characteristic** | **Total (n=125)** | **TDM (n=44)** | **Control (n=81)** | **P-value** |
| --- | --- | --- | --- | --- |
| Age, y | 59 (+15) | 61 (+16) | 57 (+15) | 0.18 |
| Age > 60 y | 68 (54) | 29 (66) | 39 (48) | 0.06 |
| Age > 65 y | 50 (40) | 23 (52) | 27 (33) | **0.04** |
| Male | 74 (59) | 28 (64) | 46 (57) | 0.46 |
| BMI, kg/m^2^ | 29.5 (+7.7) | 29.3 (+7.7) | 29.6 (+7.8) | 0.88 |
| Serum creatinine, mg/dL | 0.82 (0.66 – 1.26) | 0.85 (0.66 – 1.38) | 0.82 (0.66 – 1.10) | 0.28 |
| CrCl, mL/min | 88 (59 – 126) | 74 (55 – 122) | 91 (62 – 126) | 0.25 |
| CrCl < 60 mL/min | 32 (26) | 14 (32) | 18 (22) | 0.24 |
| CrCl < 30 mL/min | 5 (4) | 3 (7) | 2 (2) | 0.24 |
| Platelets, K/mcL | 315 (+134) | 303 (+146) | 321 (+128) | 0.47 |
| Surgical procedure | 21 (17) | 13 (30) | 8 (10) | **0.005** |
| COpAT monitoring | 25 (20) | 19 (43) | 6 (7) | **< 0.001** |
| Receipt of other antibiotics | 65 (52) | 23 (52) | 42 (52) | 0.96 |
| Infection type* | | | | |
| Bone and joint | 77 (62) | 24 (55) | 53 (65) | 0.23 |
| Skin and soft tissue | 20 (16) | 5 (11) | 15 (19) | 0.30 |
| Intra-abdominal | 13 (10) | 5 (11) | 8 (10) | 0.77 |
| Endovascular | 11 (9) | 7 (16) | 4 (5) | **0.05** |
| Bloodstream | 10 (8) | 4 (9) | 6 (7) | 0.74 |
| Respiratory | 4 (3) | 3 (7) | 1 (1) | 0.13 |
| Central nervous system | 3 (2) | 1 (2) | 2 (2) | 1.00 |
| Other | 9 (7) | 2 (5) | 7 (9) | 0.40 |
| Initial planned duration of linezolid | | | | |
| 21-27 d | 23 (18) | 6 (14) | 17 (21) | 0.31 |
| 28-41 d | 83 (66) | 31 (70) | 52 (64) | 0.48 |
| > 42 d | 19 (15) | 7 (16) | 12 (15) | 0.87 |
| Linezolid duration inpatient, d | 5.1 (+6.3) | 8.4 (+8.6) | 3.4 (+3.7) | **< 0.001** |
| Linezolid duration outpatient, d | 27.4 (+11.9) | 26.0 (+13.8) | 28.2 (+10.8) | 0.33 |
| Total linezolid duration, d | 31.6 (+12.1) | 33.7 (+14.0) | 30.7 (+10.9) | 0.19 |
| Weekly outpatient CBCs | 59 (47) | 26 (59) | 33 (41) | **0.05** |
| Total outpatient CBCs drawn | 2.7 (+1.6) | 2.9 (+1.8) | 2.6 (+1.5) | 0.28 |
| Continuous variables are reported as means (+SD) if parametric and medians (IQR) if non-parametric, frequencies are reported as n (%). Demographics and laboratory parameters are reported at time of linezolid initiation. BMI, Body mass index; CBC, Complete blood count; COpAT, Complex outpatient antimicrobial therapy; CrCl, Creatinine clearance; PP, per-protocol; TDM, Therapeutic drug monitoring.  *Multiple infection types may co-present, and both are reported individually (e.g. osteomyelitis with overlying cellulitis is both bone and joint and skin and soft tissue). | | | | |

**Table S2. Demographics and baseline characteristics of patients receiving linezolid TDM by PP status**

| **Characteristic** | **All TDM (n=81)** | **PP (n=44)** | **Non-PP (n=37)** | **P-value** |
| --- | --- | --- | --- | --- |
| Age, y | 60.7 (+16.0) | 61.1 (+15.7) | 60.3 (+16.5) | 0.84 |
| Age > 60 y | 50 (62) | 29 (66) | 21 (57) | 0.40 |
| Age > 65 y | 42 (52) | 23 (52) | 19 (51) | 0.93 |
| Male | 46 (57) | 28 (64) | 18 (49) | 0.17 |
| BMI, kg/m^2^ | 28.6 (+7.4) | 29.3 (+7.7) | 27.8 (+7.1) | 0.34 |
| Serum creatinine, mg/dL | 0.86 (0.67 – 1.26) | 0.85 (0.66 – 1.38) | 0.86 (0.67 – 1.05) | 0.50 |
| CrCl, mL/min | 81 (59 – 104) | 74 (55 – 122) | 83 (63 – 100) | 0.79 |
| CrCl < 60 mL/min | 21 (26) | 14 (32) | 7 (19) | 0.21 |
| CrCl < 30 mL/min | 4 (5) | 3 (7) | 1 (3) | 0.62 |
| Platelets, K/mcL | 297 (+132) | 303 (+146) | 289 (+116) | 0.63 |
| Surgical procedure | 23 (28) | 13 (30) | 10 (27) | 0.80 |
| COpAT monitoring | 25 (31) | 19 (43) | 6 (16) | **0.01** |
| Receipt of other antibiotics | 49 (60) | 23 (52) | 26 (70) | 0.10 |
| Infection type* | | | | |
| Bone and joint | 47 (58) | 24 (55) | 23 (62) | 0.49 |
| Skin and soft tissue | 14 (17) | 5 (11) | 9 (24) | 0.15 |
| Intra-abdominal | 10 (12) | 7 (16) | 3 (8) | 0.33 |
| Endovascular | 7 (9) | 5 (11) | 2 (5) | 0.45 |
| Bloodstream | 6 (7) | 4 (9) | 2 (5) | 0.68 |
| Respiratory | 3 (4) | 3 (7) | 0 (0) | 0.25 |
| Central nervous system | 1 (1) | 1 (2) | 0 (0) | 1.00 |
| Other | 4 (5) | 2 (5) | 2 (5) | 1.00 |
| Initial planned duration of linezolid | | | | |
| 21-27 d | 17 (21) | 6 (14) | 11 (30) | 0.10 |
| 28-41 d | 52 (64) | 31 (70) | 21 (57) | 0.20 |
| > 42 d | 12 (15) | 7 (16) | 5 (14) | 1.00 |
| Linezolid duration inpatient, d | 6.5 (+7.0) | 8.4 (+8.6) | 4.2 (+3.4) | **0.007** |
| Linezolid duration outpatient, d | 28.1 (+13.9) | 26.0 (+13.8) | 30.5 (+13.8) | 0.14 |
| Total linezolid duration, d | 33.7 (+13.6) | 33.7 (+14.0) | 33.7 (+13.4) | 0.96 |
| Weekly outpatient CBCs | 42 (52) | 26 (59) | 16 (43) | 0.16 |
| Total outpatient CBCs drawn | 3.1 (+1.9) | 2.9 (+1.8) | 3.4 (+2.1) | 0.31 |
| Continuous variables are reported as mean (+SD) if parametric and median (IQR) if non-parametric, frequencies are reported as n (%). Demographics and laboratory parameters are reported at time of linezolid initiation. BMI, Body mass index; CBC, Complete blood count; COpAT, Complex outpatient antimicrobial therapy; CrCl, Creatinine clearance; PP, per-protocol; TDM, Therapeutic drug monitoring.  *Multiple infection types may co-present, and both are reported individually (e.g. osteomyelitis with overlying cellulitis is both bone and joint and skin and soft tissue). | | | | |

**Table S3.** Incidence of alternative causes of early discontinuation by use of linezolid TDM in the matched and PP populations

| **Outcome** | **Total** | **TDM** | **Control** | **P-value** |
| --- | --- | --- | --- | --- |
| **Matched population** | **n=162** | **n=81** | **n=81** |  |
| Early discontinuation not due to thrombocytopenia | 21 (13) | 14 (17) | 7 (9) | 0.10 |
| Adverse event* | 14 (9) | 9 (11) | 5 (6) | 0.40 |
| Other clinical reason** | 7 (4) | 5 (6) | 2 (2) | 0.44 |
| **PP population** | **n=125** | **n=44** | **n=81** |  |
| Early discontinuation not due to thrombocytopenia | 17 (10) | 10 (23) | 7 (9) | **0.05** |
| Adverse event | 11 (7) | 6 (14) | 5 (6) | 0.19 |
| Other clinical reason | 6 (4) | 4 (9) | 2 (2) | 0.18 |
| Continuous variables are reported as mean (+SD), frequencies are reported as n (%). PP, per-protocol; TDM, Therapeutic drug monitoring.  *Adverse event reported, **TDM arm:** gastrointestinal symptoms (n=3), anemia (n=1), dizziness (n=1), leukopenia (n=1), peripheral neuropathy (n=1), rash (n=1), transaminitis (n=1); **non-TDM arm:** bruising (n=1), facial swelling (n=1), fatigue (n=1), gastrointestinal symptoms (n=1), leukopenia (n=1).  **Other clinical reason, **TDM arm:** death/hospice (n=2), duration shortened due to improvement (n=2), therapy changed upon readmission (n=1); **non-TDM arm:** lack of improvement (n=2). | | | | |

**Table S4.** Non-thrombocytopenia adverse events reported during linezolid treatment in the matched population

| **Adverse event** | **TDM** | **Control** |
| --- | --- | --- |
| Gastrointestinal symptoms | 13 (16) | 12 (15) |
| Fatigue | 5 (6) | 4 (5) |
| Poor taste/appetite | 5 (6) | 2 (2) |
| Anemia | 2 (2) | 1 (1) |
| Dizziness | 2 (2) | 0 (0) |
| Leukopenia | 1 (1) | 1 (1) |
| Peripheral neuropathy | 1 (1) | 1 (1) |
| Acidosis | 0 (0) | 1 (1) |
| Bruising | 0 (0) | 1 (1) |
| Chest pain | 1 (1) | 0 (0) |
| Facial edema | 0 (0) | 1 (1) |
| Hypoglycemia | 0 (0) | 1 (1) |
| Mouth blisters | 0 (0) | 1 (1) |
| Photosensitivity | 1 (1) | 0 (0) |
| Rash | 1 (1) | 0 (0) |
| Transaminitis | 1 (1) | 0 (0) |
| Vision changes | 1 (1) | 0 (0) |
| Frequencies are reported as n (%). TDM, Therapeutic drug monitoring. | | |
